# Polish translation, cross-cultural adaptation, and psychometric evaluation of the Frommelt Attitude Toward Care of the Dying Scale — Form A (FATCOD-A) among nurses

**DOI:** 10.1186/s12912-025-03994-x

**Published:** 2025-10-29

**Authors:** Patrycja Krężel, Magdalena Kuczyńska, Joanna Owsianowska, Sylwia Wieder-Huszla, Anna Jurczak

**Affiliations:** 1 Department of Specialized Nursing, Pomeranian Medical University in Szczecin, Szczecin, 71-210 Poland; 2 Department of Social Medicine, Subdepartment of Long-Term Care and Palliative Medicine, Pomeranian Medical University in Szczecin, Szczecin, 71-210 Poland

**Keywords:** Death, Palliative care, Validation study, Attitude, FATCOD A

## Abstract

**Background:**

Nurses’ attitudes towards death, dying patients and End-of-Life care have a great significance for the quality of provided care. Various factors influence nurses’ attitudes towards death. Negative attitudes or ineffective ways of coping with stress caused by patient’s death can lead to severe implications, such as job burnout or avoidance and hostility toward dying patients. Therefore, evaluation of nurses’ attitudes towards caring for terminally ill is essential for providing good and suitable palliative care.

**Methods:**

The aim of the study was to validate and culturally adapt the Frommelt Attitude Toward Care of the Dying Scale Form A. Research was conducted among 283 polish nurses with the use of questionnaire translated by two independent certified interpreters. Polish version of the tool was accepted by the author of the scale - K. H. M. Frommelt. The reliability of the scale was calculated using Cronbach’s Alpha coefficient. The validity analysis was determined by assessing the correlation between sociodemographic data and the results of the scale. Factor analysis was performed using the principal component analysis and Varimax rotation method.

**Results:**

The analysis revealed two-factorial structure of the tool. Internal consistency of the scale calculated with the Cronbach’s Alpha coefficient showed adequate reliability of the entire scale (α = 0,820) and its factors (α = 0.808 and α = 0.757 respectively). The final version of the Polish scale consists of 30 items. Also, the study revealed correlation between nurses’ attitude and gender, age, workplace, work experience and education.

**Conclusions:**

This preliminary study demonstrated good internal consistency and a two-dimensional structure of Polish version of FATCOD A, and the results suggest that further validation work should be performed. It is important to expand the database of tools designed to conduct research among nurses and this study provides another instrument that can be used for this purpose.

**Supplementary Information:**

The online version contains supplementary material available at 10.1186/s12912-025-03994-x.

## Background

 Death is an inevitable part of every existence. For centuries, people have tried to understand its nature and have analyzed its various aspects - biological, religious, social, and philosophical. However, medicine and its related sciences require focusing on the physical aspect of death and how it should be defined [1]. There are many definitions of death, but the most common one is that a person is considered dead when there is a complete and irreversible cessation of the brain function. Death may occur e.g. as a result of traumatic brain injury or lack of blood supply to the brain [2].

Death can have different forms - it can occur suddenly or be a process extended over time. Every patient’s death is different, and the circumstances under which it occurred will evoke all sorts of emotions. Process of dying is always a stressful event and has a negative impact on everyone involved – the patient, their relatives, and nursing staff providing End-of-Life care. Nurses often develop an emotional bond with patients and guiding the terminally ill through the final stage of life can cause feelings of distress, uncertainty, grief, and defeat among healthcare professionals [3–7]. Furthermore, depending on whether death is a predictable event or a sudden one, it can create different challenges for the nursing staff. In the event of a sudden deterioration of the patient’s condition, nurses may experience stress related to constantly monitoring the patient’s parameters and providing life-saving interventions. In addition, if the patient requires a transition from curative to End-of-Life (EoL) care, it may further intensify the negative emotions and often be associated with a moral dilemma [4, 5, 8, 9]. For patients who are slowly approaching the end of life, care aims to alleviate the symptoms associated with their illness and to guide them through the dying process as gently as possible. In order to ensure that the patient has a peaceful death, it is necessary to have the right approach to this difficult subject and to treat the patient with calmness and patience [7]. End-of-life care requires a lot of empathy from healthcare professionals - the ability to talk to the patient and their family about the impending death, the skills to manage and control one’s emotions and to remain professional in every situation. Providing palliative care is a process extended in time, and during that time various challenges may arise. Terminally ill patients, as well as their families, are often overwhelmed with uncertainty and fear. Care and emotional support should therefore be provided to both the patient and their loved ones, which also puts additional pressure on the nurse and leads to challenging conversations. Many nurses, especially those working in emergency units, admit that they often lack the ability to discuss death and the process of dying with the patients [5, 10, 11]. Furthermore, EoL care is focused on guiding the patient through the last stage of life, rather than saving it, and can therefore cause feelings of resignation, helplessness, emotional exhaustion, and discouragement [10, 12]. The ability to provide palliative care is becoming increasingly needed due to the institutionalization of death. It is currently observed that the number of deaths in nursing homes and long-term care institutions is raising [13, 14]. Still the preferred place of death for most patients is at home, but the patient’s condition does not always allow them to pass away in their own bed, and being in a nursing home helps to access continuous professional care [15, 16].

Regardless of how the patient dies and whether this death takes place in a hospital ward, hospice, nursing home or other institution, there are always medical professionals participating in the process. Nurses play a huge role in caring for patients in the last stage of life - both in emergency and palliative care. The nurse is closest to the patient and spends the most time with them [17, 18]. Regardless of the workplace of the nurse, an encounter with the death of a patient is inevitable. It is a difficult experience, and depending on many factors such as age, workplace, religion, education, culture, etc., which can affect the perception of death and the attitude towards dying patients. For instance, research shows that younger nurses with less experience are more likely to have negative attitudes towards death and are more reluctant to provide EoL care. Experience in palliative care also has a significant impact on attitudes towards death and the level of stress associated with the patient’s passing. The age of the dying patient also plays a significant role, and nurses report that it is much more difficult to come to terms with the death of a young patient [6, 10, 12, 19, 20]. A common problem that nurses face is the difficulty in discussing death, the dying process and other death-related issues with patients and their relatives. Sometimes if the subject of death makes the nurse feel anxious, they will tend to depersonalize the patient and show indifference towards them [17, 21]. Furthermore, the reaction of the patient’s relatives to the information that member of their family is dying is often unpredictable. It is not uncommon for relatives to refuse to accept the impending death of a family member, which significantly hinders the communication process and the implementation of EoL care [22].

The way a nurse approaches a dying patient, and their family will have an impact on the quality of EoL care they provide. If nursing staff is not adequately prepared to implement palliative care and is unable to appropriately discuss the issue of death, they will not be able to provide the necessary support and assistance to the dying patient and their family. Skillful provision of EoL care can improve the psychological state, facilitate the grieving process, and reduce the risk of post-traumatic stress disorder in families of the dying patients [23]. Learning how to competently approach the issue of death and gaining experience in palliative care can also help medical professionals better control and manage their emotions. Furthermore, a positive attitude towards death and dying patients can prevent burnout and desensitization to patient death [24]. Therefore, it is important to study nurses’ attitudes towards death, palliative care, and terminally ill patients. In order to assess this adequately, it is beneficial to use a reliable tool. Frommelt Attitude Toward Care of the Dying Scale is a questionnaire used worldwide and proven to be valid. In addition, FATCOD Form A was designed specifically for conducting research among nurses. To the authors’ knowledge, this version of the questionnaire has not yet been validated in Poland. An increasing number of scales designed for conducting research among nurses are being developed, therefore, the aim of this study was to conduct Polish adaptation and validation of the Frommelt Attitude Toward Care of the Dying Scale Form A, to further expand the range of tools available for conducting research in this group of medical professionals.

## Methods

### Study design and population

A diagnostic survey method was applied using a self-designed questionnaire and FACTOD A Scale translated into Polish. The translation was done by two independent translators - the first translator was an academic teacher conducting English language classes for Polish-speaking students of medical faculties at the Pomeranian Medical University. The second Polish translation was done by a sworn interpreter that provides medical and technical document translations. Both versions were then unified and approved by a panel of experts specializing in nursing and palliative care. The unified Polish version was retranslated back into English by a sworn interpreter and approved by the author. Contact with the author of the original scale was maintained throughout the study, and her consent was obtained to proceed with further steps of the research, as well as the approval of the final Polish version of the scale and permission for its use for scientific purposes by the Pomeranian Medical University. Prior to the actual study, a pilot survey was carried out in a group of 32 respondents to verify the accuracy and comprehensibility of the questions and to make any necessary adjustments to the questionnaire. After the pilot study, changes were made to the abbreviations of the responses, as participants reported difficulties in answering due to incomprehensibility of the acronyms. The survey was conducted from December 2019 to February 2020, and data was collected online. The study was conducted in accordance with the Declaration of Helsinki, and the protocol was approved by the Bioethical Commission of Pomeranian Medical University in Szczecin (KB-0012/147/05/19, May 22, 2019). All respondents were given written information and gave informed consent to take part in the study after being made aware of the purpose of the research, complete anonymity of the survey and that the collected data would be used for scientific purposes, as well as the possibility of discontinuing participation in the study at any time. The assumed number of respondents was 10 people per item, i.e. 300 people. A total of 297 questionnaires were obtained, but 14 were rejected due to incorrect completion or incomplete answers. The questionnaire was made available via social media – in closed groups for nurses that required confirmation of practicing this profession. The sample is large enough that it was decided that it was unnecessary to check the normality of the variable distributions. It can be assumed in advance that parametric tests and coefficients will be appropriate. The same applies to the homogeneity of variance. The criteria for inclusion in the study were working as a nurse, consenting to participate in the study, and actively working in the profession. Exclusion criteria were age under 18, not being a professional nurse, not agreeing to participate in the study and not completing the questionnaire correctly.

### Research instruments

The tools used in this study were the Frommelt Attitude Toward Care of the Dying Scale Form A created by Katherine H. M. Frommelt and a self-designed questionnaire collecting sociodemographic and workplace data, frequency of interactions with dying patients and their families, and level of received palliative care education [25].

#### Frommelt Attitude Toward Care of the Dying Scale Form A (FATCOD – A)

FATCOD is a scale used to evaluate the attitude of nursing staff toward the care of terminally ill patients and their families. The questionnaire consists of 30 statements − 15 of which are positive and the other 15 are negative. Positive statements are scored from 1 (strongly disagree) to 5 (strongly agree). For negative statements, points are counted in reverse. The lowest score that can be obtained is 30, and the highest is 150. The higher the score, the more positive the nurse’s attitude toward caring for dying patients is [25, 26]. In this study, a two-factor structure of the tool was found, and it was observed that Factor 1 included more negatively worded items while Factor 2 had more positively worded or family related statements, but items 2 and 29 did not meet the criteria for allocation to the given factors and could not be included in any of the dimensions.

### Data analysis

The obtained results were subjected to statistical analysis. Reliability was calculated using Cronbach’s Alpha coefficient. Factor analysis was also carried out - performed by the principal component analysis, initially applying a Varimax rotation using the principal component analysis for all questions, with no number of components assumed in advance with cut-off used to retain item being > 0,400. A Kaiser-Mayer-Olkin (KMO) measure of sampling adequacy and a Barlett’s test of sphericity were also performed. A scatter plot was used as well, and the sums of squared loadings were calculated to determine the number of factors. Lastly, a scale validity analysis was performed. It involved examining the correlations between sociodemographic characteristics and the results obtained from the questionnaire. The data from the analysis was compared with assumptions regarding how demographic characteristics should influence the results. Confirmation of these assumptions proves the accuracy of the tool. For this purpose, the Student’s t-test for independent groups, analysis of variance (ANOVA), Tukey’s multiple comparisons test and Pearson’s correlation coefficient (r) were used. The sample was considered large enough that the normality of distribution and homogeneity of variance were not performed, assuming in advance that parametric tests and coefficients would be appropriate. The analysis was performed in IBM SPSS Statistics 26.

## Results

### Demographic characteristics

The study group comprised of 283 nurses, with the group being predominantly female (96%). The mean age of the surveyed nurses was 40.1 years (SD 11.19). The majority of the nursing staff had a higher education - either bachelor’s (46%) or master’s degree level (44%). Most frequently, respondents were employed in non-invasive treatment wards, which do not provide surgical treatment and are often referred to as internal medicine wards (43%). As many as 86% of participants declared frequent contact with dying patients, but only 23% had a course or specialization in palliative care. The average length of professional experience was 16.8 years (SD 12.2). In the study group, the mean FATCOD score was 114.8 (SD 11.7). The respondents in the vast majority showed a positive attitude (91.9%). Only a small percentage of respondents obtained scores corresponding with an intermediate attitude (8,1%), while none of the surveyed nurses showed a negative attitude.

### Reliability

The reliability of the Polish version of FATCOD A was obtained using the Cronbach’s Alpha coefficient. The value calculated for the entire scale containing all 30 items was 0.820. Item 2 showed a weak correlation, but after consulting with a statistician and evaluating the content of the question, it was decided to retain the item. Its removal would only slightly improve the reliability of the scale (from 0.820 to 0.830). Furthermore, it is the only item referring to the general attitude towards death. Therefore, it was not considered appropriate to remove this item from the scale.

### Factor analysis

First, a factor analysis with Varimax rotation was performed using the principal component analysis method for all questions, without assuming any number of components in advance. The Kaiser-Mayer-Olkin test of sampling adequacy was 0.818, and the Barlett’s test of sphericity Chi-2 = 181,777; df = 435; *p* < 0.001, meaning that it was possible and valid to perform a factor analysis. Each item showed a value above 0.400, which means that all statements are suitable for factor analysis. The results are shown in Table 1.

**Table 1:** Communalities of individual items after extraction using the principal component analysis method with varimax rotation

FACTOD item number	After extraction using the principal component method with Varimax rotation
P1	0,607
P2	0,688
P3	0,518
P4	0,629
P5	0,541
P6	0,609
P7	0,468
P8	0,518
P9	0,521
P10	0,581
P11	0,609
P12	0,505
P13	0,543
P14	0,620
P15	0,516
P16	0,563
P17	0,584
P18	0,636
P19	0,406
P20	0,630
P21	0,498
P22	0,605
P23	0,646
P24	0,595
P25	0,572
P26	0,474
P27	0,546
P28	0,666
P29	0,613
P30	0,525

**Fig. 1:**
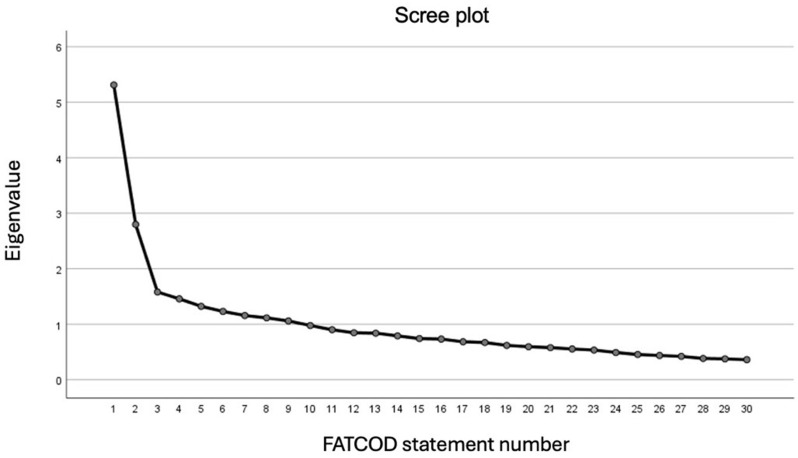
Scatterplot for the determination of a number of factors of the Polish FATCOD A scale

**Table 2:** Sums of squares of squared loadings

Compontent	Sums of squares of squared loadings		
	Overall	% of variance	Cumulative %
1	3,225	10,751	10,751
2	2,418	8,059	18,810
3	1,925	6,418	25,227
4	1,820	6,067	31,295
5	1,779	5,931	37,225
6	1,618	5,394	42,619
7	1,480	4,932	47,552
8	1,464	4,879	52,431
9	1,300	4,332	56,763

The Polish version of FATCOD A was found to be two-factorial. In order to determine the number of factors, a scatterplot test was performed and the values of sums of squared loadings were calculated, which showed the existence of two components. When calculating the sum of squared loadings, nine scale components were taken into account, due to the fact that they were the only items that met the criteria for these calculations. The values for the first two components are significantly higher than for the subsequent ones. This also supports a two-factor solution. These finding are presented in Fig. 1 and Table 2.

Factor analysis was then performed again, this time including a two-factor solution. A dark colour means that an item can be undoubtedly included in a specific factor. A slightly lighter colour indicates that the difference between the item loadings is not clear but it can still be included in a specific component. Statements 2 and 29 did not meet the criteria for allocation to the given factors and could not be included in any of the dimensions. However, they were not removed from the scale. Item 2 was retained due to its meaning – ‘Death is not the worst thing that can happen to a person’ – which is a statement referring to death in general, rather than to the patient or their family, which supports not assigning this item to any of the factors. Item 29 - ‘Family members who stay close to a dying person often interfere with the professionals’ job with the patient’ was left on the scale due to its good correlation with the scale as a whole and the importance of the matter that is contained in this item.

The breakdown of the factors is as follows:

Factor 1: 1,3,5,6,7,8,9,11,13,14,15,17,26,28.

Factor 2: 4,10,12,16,18,19,20,21,22,23,24,25,27,30.

The above data are presented in Table 3.

**Table 3:** Factor loadings of FATCOD A scale items

FATCOD item number	Factor	
	1	2
P1	0,323	0,175
P2	0,128	-0,053
P3	0,579	-0,053
P4	0,155	0,414
P5	0,657	0,032
P6	0,625	0,157
P7	0,572	0,048
P8	0,379	-0,050
P9	0,419	0,084
P10	0,090	0,366
P11	0,529	0,230
P12	-0,022	0,437
P13	0,637	0,106
P14	0,613	0,070
P15	0,626	0,202
P16	0,061	0,650
P17	0,383	0,295
P18	-0,087	0,679
P19	0,122	0,442
P20	0,032	0,619
P21	0,227	0,393
P22	0,084	0,595
P23	0,003	0,557
P24	0,000	0,525
P25	0,024	0,361
P26	0,599	-0,018
P27	0,259	0,377
P28	0,309	0,084
P29	0,259	0,167
P30	0,335	0,363

It was observed that the items belonging to Factor 1 coincide with the negative statements and the items from Factor 2 with those worded positively and relating to the attitude towards patient’s family. A reliability analysis of both factors using Cronbach’s Alpha coefficient was also performed. For Factor 1, the Cronbach’s Alpha coefficient was 0.808 and for Factor 2 it was 0.757.

### Validity analysis

The validity analysis involved conducting an exploratory factor analysis (EFA) determining the correlation between sociodemographic data and the results of the scale. The Student’s t-test for independent groups was used to assess the correlations between attitudes towards care of the dying patients and gender and place of work. The analysis showed that there was a statistically significant difference in terms of gender in relation to Factor 2 - women were more likely to have a positive attitude towards care of terminally ill patients than men. In addition, the analysis between nurses employed and non-employed on surgical wards showed a statistically significant difference for Factor 1 and the total score. Similar results were obtained when comparing nursing staff working and non-working in hospice - a significant correlation was shown for Factor 1 and the total score, meaning that those working in hospice showed significantly more positive attitudes in relation to Factor 1 and the total score. The results are presented in Table 4.

**Table 4:** Correlations between FATCOD A results and gender and workplace

FATCOD score	Variable				Student’s t-test	
	Gender					
	Female		Male			
	M	SD	M	SD	t	*p*
Factor 1	49,42	8,16	50,25	7,06	-0,348	0,728
Factor 2	58,95	5,47	55,50	5,84	2,133	0,034*
Overall score	114,70	11,76	112,25	11,15	0,709	0,479
	Surgical ward					
	Yes		No			
	M	SD	M	SD	t	p
Factor 1	47,21	7,00	50,45	8,38	-3,151	0,002*
Factor 2	58,40	5,68	58,98	5,45	-0,812	0,417
Overall score	111,94	10,81	115,78	11,95	-2,565	0,011*
	Hospice					
	Yes		No			
	M	SD	M	SD	t	p
Factor 1	53,47	7,52	48,65	8,00	3,804	< 0,001*
Factor 2	60,17	5,37	58,53	5,51	1,871	0,062
Overall score	120,09	11,18	113,51	11,55	3,583	< 0,001*

M – mean, SD – standard deviation, t – Student’s t, *p* < 0,05

To analyze the correlation between education, including palliative care education, and the FATCOD score, analysis of variance (ANOVA) and the Tukey multiple comparison test were used. For education, which was divided into 3 groups (1 - nursing secondary school, 2 -bachelor’s degree, 3 - master’s degree), the analysis showed a statistically significant difference for Factor 2 and total FATCOD score. Considering Factor 2, those in groups 2 and 3 showed a significantly higher score than respondents in group 1. Meanwhile, there are no significant differences between groups 2 and 3. On the other hand, group 3 has a higher overall score than group 1. For palliative care education, 3 groups were also formed (1 - nurses with a course or specialization in palliative care; 2 - staff with courses or specializations in another field; 3 - nursing personnel with no further education). Statistically significant correlations were shown for both factors and the total FATCOD score. Considering Factor 1, those in group 1 showed a significantly higher score than those in groups 2 and 3. However, no significant differences were observed between groups 2 and 3. The data look similar for the total score. For Factor 2, group 1 showed a significantly higher score than those in group 3. The data described above are shown in Table 5.

**Table 5:** Correlation between FATCOD score and academic background and palliative care education

FATCOD score	Variable						ANOVA		Tukey Test
	Level of education								
	Nursing secondary school (1)		Bachelor’s degree (2)		Master’s degree (3)				
	M	SD	M	SD	M	SD	F	*p*	
Factor 1	47,48	9,18	48,88	7,82	50,50	8,07	2,226	0,110	
Factor 2	55,34	4,65	58,69	5,65	59,72	5,26	7,817	< 0,001*	1 < 2,3
Overall score	109,00	12,26	113,97	11,24	116,55	11,70	5,386	0,005*	1 < 3
	Courses/specialization								
	Palliative care		Other		None		F	p	
	M	SD	M	SD	M	SD			
Factor 1	52,98	7,79	48,82	7,95	47,81	7,89	8,877	< 0,001*	1 > 2,3
Factor 2	60,35	5,41	58,40	5,52	58,25	5,44	3,417	0,034*	1 > 3
Overall score	119,63	11,39	113,54	11,84	112,48	10,86	8,395	< 0,001*	1 > 2,3

For the analysis of respondents’ age and years of work experience, linear correlations were examined between the variables. Therefore, Pearson’s correlation coefficient (r) was calculated. The analysis showed that both age and years of work experience showed a statistically significant correlation, but only for Factor 2. The higher the age and the longer the work experience, the less positive the attitude expressed in Factor 2. The results are shown in Table 6.

**Table 6:** Correlation between FATCOD score and age and years of work experience

FATCOD score	Age		Work experience	
	*r*	*p*	*r*	*p*
Factor 1	-0,030	0,612	-0,065	0,279
Factor 2	-0,143	0,016*	-0,160	0,007*
Overall score	-0,086	0,150	-0,114	0,055

r - correlation coefficient, *p* < 0,05

## Discussion

Contact with death is part of the nursing profession. Every nurse will have to, at least once, provide care for a dying patient, whether their death is sudden or expected. Therefore, it is important to assess nurses’ attitudes not only towards death itself, but also towards dying patients and their families. There are two versions of the Frommelt Attitude Toward Care of the Dying Scale - Form A and Form B. Validation and adaptation of the FATCOD - B scale has already been carried out in Poland and can be used among various medical professionals [27]. However, the authors of this study believe it is important that the FATCOD A should also be available for use in Poland because of its applicability specifically in the nursing community.

The FATCOD scale has been validated in many countries, adaptations have been carried out and very different results have been obtained regarding the structure of the tool, the number of factors, as well as the reliability and validity values of the scale.

The structure of the FATCOD scale differs between studies. Researchers report obtaining varying numbers of factors. For non-shortened versions of the scale, two factors are most commonly distinguished [27–30]. However, there have been studies that have found FATCOD to have many more dimensions. Three factors were distinguished in Vietnamese version, six factors in Turkish adaptation or even seven-factor structure in Chinese research. In these studies, EFA analysis was performed to determine the number of factors. Cultural influences may be related to the number of distinguished factors [17, 31, 32]. In this study, a two-factor structure of the tool was found.

Additionally, it is not uncommon for some items to be unsuitable for inclusion in any of the dimensions. In this study items 2 and 29 did not meet the criteria for allocation to any given factors and could not be included in any of the dimensions. Similar observations were made in the study by Leombruni P., et al. - where 7 statements did not have an adequate factor loading, including items 2 and 29. In the Polish study examining Form B of the FATCOD scale, three items, two of which were items 2 and 29, were also not included in any of the factors [27, 29].

There are also differences in the number of items in validated versions of the FATCOD scale - originally, the FATCOD scale comprises of 30 items, but in various studies it was necessary to remove certain items from the scale often due to the low factor loading. The number of items not meeting the statistical requirements varied - some studies removed only two items, and in other research as many as 13 items had very weak loading [17, 31]. Depending on the country, different items were found to be problematic. For example, in the Swedish study, items 1, 2, 10, 23, 25, and 30 significantly lowered Cronbach’s alpha, and their removal would improve the reliability of the scale [28]. In this study, the removal of item 2 was considered due to its poor correlation but discarding it would not have significantly improved the reliability of the scale (from 0.820 to 0.830). With the overall reliability of the scale being considerably high, it was decided to retain the statement.

In the various adaptations, differences in scale reliability can also be observed. The scores obtained are dispersed over a wide range. Some relatively low reliability values were found in a study conducted in Italy, where the Cronbach’s alpha coefficient was 0.699, or in Sweden, where, after removing six statements, the Cronbach’s alpha was 0.70 [28, 29]. The most common Cronbach’s Alpha values obtained in many studies ranged between 0.725 and 0.79 for the whole scale [17, 27, 29, 32–34]. In the own study, the Cronbach’s alpha coefficient was relatively high with a value of 0.820.

It is also worth noting that studies have also been carried out with the aim of shortening the FATCOD scale - such research has already been done in Italy and Sweden, where both studies have developed scales with 9 items each with good reliability and validity. This is an option worth considering for its simplicity of use and faster scoring [35, 36].

### Limitations

The study had some limitations. First of all, the group was predominantly female and mainly had higher education (bachelor’s and master’s degrees), which may have influenced the results. A less homogeneous group in terms of education and gender should be selected. In addition, the survey was conducted via an online survey. This form of administering the questionnaire may have impacted the results of the study. Furthermore, the study was conducted among nurses with varying levels of experience in caring for dying patients. It would be reasonable to conduct research on a group of nurses exclusively involved in providing palliative care. It should also be noted that confirmatory factor analysis (CFA) was not performed, which is an important element of tool validation. However, this was a preliminary study, and statistical calculations, including CFA, will be expanded in subsequent research. It was also impossible to perform test-retest reliability due to the inability to recontact the study participants. In future studies, the questionnaire will be administered in various centers providing care for terminally ill patients, which will make it feasible to reach the respondents once again and perform test-retest reliability.

#### Implication for practice

Although the study is preliminary, it can be assumed that the Polish version of the FATCOD-A scale proves to be a useful tool for conducting research among nursing staff - it expands the base of questionnaires designed specifically for conducting research among nurses and the questionnaire can be used in assessment of training of nursing staff (e.g. before and after palliative care classes, specializations, postgraduate studies in terminal/palliative care).

#### Future research

It would be advisable to conduct another study using the FATCOD-A scale in order to expand the statistical calculations and perform CFA in independent samples and verify test-retest reliability. In future studies, shortening the questionnaire could also be considered.

## Conclusions

The Polish FATCOD-A demonstrates acceptable internal consistency (α = 0.82) and an interpretable two-factor structure in this exploratory sample of nurses (*n* = 283). These preliminary psychometric results support further validation work, including confirmatory factor analysis and test–retest reliability in independent samples. Polish version of FATCOD – A has 30 items - the same as the original version of the questionnaire. The scale consists of two factors - Factor I, which includes mostly negatively worded statements, and Factor II, which includes mainly positively worded items and those referring to the attitude towards the patient’s family. The analysis also showed their high internal consistency. Although a validated version of the FATCOD-B already exists in Poland, the authors of this study believe that it is important that the FATCOD-A scale is also available to Polish researchers given its suitability for studies specifically involving nursing staff. Currently, an increasing number of questionnaires are being developed which are intended to be used only to conduct research among nurses. Therefore, the authors believe that it is worth expanding the Polish database of tools for research in this particular group of professionals allowing for more complex studies in the future.

## Supplementary Information

Below is the link to the electronic supplementary material.

Supplementary Material 1: FATCOD – English version of questionnaire guide. The original contributions presented in the study are included in the article and its supplementary information files, and further inquiries can be directed to the corresponding authors.

## Data Availability

The original contributions presented in the study are included in the article and its supplementary information files. Any other dataset is available from the corresponding author on reasonable request.

## Abbreviations

FATCOD: Frommelt Attitude Toward the Care of the Dying Scale
EoL: End–of–life
KMO: Kaiser–Meyer–Olkin test
ANOVA: Analysis of Variance
EFA: Exploratory factor analysis
CFA: Confirmatory factor analysis
