# Supplementary material for: Polish translation, cross-cultural adaptation, and psychometric evaluation of the Frommelt Attitude Toward Care of the Dying Scale — Form A (FATCOD-A) among nurses
Source: BMC Nurs. 2025 Oct 29;24:1343. doi: 10.1186/s12912-025-03994-x (PMC12570458; doi:10.1186/s12912-025-03994-x)
Supplement: Supplementary file 1 — Supplementary Material 1: FATCOD – English version of questionnaire guide. The original contributions presented in the study are included in the article and its supplementary information files, and further inquiries can be directed to the corresponding authors. [file 12912_2025_3994_MOESM1_ESM.docx]

**Frommelt Attitude Toward Care of the Dying Scale**

**Form A –English translation of Polish FATCOD version**

The purpose of the following statements is to learn how nursing staff feels about certain situations in which they are involved in patients. All statements concern providing care to a dying person and/or his/her family. In the following questionnaire, a dying patient should be considered as a terminally ill person with a life expectancy of six month or less.

Please circle the letter following each statement which corresponds to your own personal feelings about the attitude or situation presented. Please respond to all 30 statements on the scale. The meaning of the letters is:

SD = Strongly Disagree

D = Disagree

U = Uncertain

A = Agree

SA = Strongly Agree

1. Giving nursing care to the dying person is a **valuable** learning experience.

SD D U A SA

2. Death is not the worst thing that can happen to a person.

SD D U A SA

3. **Talking about impeding death with the dying person would make me uncomfortable.**

SD D U A SA

4. Nursing care for the patient's family should continue throughout the period of grief. SD D U A SA

5. I would not want to be assigned to care for a dying person.

SD D U A SA

6. The nurse should not be **the person assigned** to talk about death with the dying person. SD D U A SA

7. The length of time required to give nursing care to a dying person would frustrate me.

SD D U A SA

8. I would be upset when the dying person I was caring for gave up hope of getting better.

SD D U A SA

9. It is difficult to form a close relationship with the family of the dying person.

SD D U A SA

10. There are times when death is welcomed by the dying person.

SD D U A SA

11. When a patient asks **if** **he/she is dying**, I think it is best to change the subject to something cheerful. SD D U A SA

12. The family should be involved in **taking** care of the dying person.

SD D U A SA

13. I would hope the patient I'm caring for dies when I am not present.

SD D U A SA

14. I am afraid to become friends with a dying person.

SD D U A SA

15. **The death of the patient I’m taking care of would make me want to run away.**

SD D U A SA

16. Families need emotional support to accept the behavior changes of the dying person.

SD D U A SA

17. As a patient nears death, the nurse should withdraw from his/her involvement **in contacts** with the patient. SD D U A SA

18. Families should be concerned about helping their dying member make the best of his/her remaining life. SD D U A SA

19. The dying person should not be allowed to make decisions about his/her **own** care.

SD D U A SA

20. **Family** should maintain as normal an environment as possible for their dying member.

SD D U A SA

21. It is beneficial for the dying person to verbalize his/her feelings.

SD D U A SA

22. Nursing Care should extend to the family of the dying person.

SD D U A SA

23. **Persons providing nursing care** should permit dying persons to have flexible visiting schedules.

SD D U A SA

24. The dying person and his/her family should be the in-charge decision makers.

SD D U A SA

25. Addiction to pain relieving medication should not be a concern when **regard to** a dying person. SD D U A SA

26. I would be uncomfortable if I entered the room of a terminally ill person and found him/her crying. SD D U A SA

27. Dying persons should be given honest answers about their condition.

SD D U A SA

28. Educating **the family** about death and the **process of** dying is not the responsibility **of a nurse.**

SD D U A SA

1. Family members who **stay in close relations** to a dying person often interfere **with providing care to the patient.**  SD D U A SA

30. It is possible for nurses to help patients prepare for death.

SD D U A SA
